# 719 Child Life Specialists in the Treatment of Acute Pediatric Burns: A Systematic Review

**DOI:** 10.1093/jbcr/irae036.263

**Published:** 2024-04-17

**Authors:** Paige Knight, Zoe Kore, Sally L Hynes

**Affiliations:** Division of Plastic Surgery, University of Birtish Columbia, Vancouver, BC; Faculty of Medicine, University of British Columbia, Vancouver, BC; Division of Pediatric Plastic Surgery, British Columbia Children's Hospital, Vancouver, BC; Division of Plastic Surgery, University of Birtish Columbia, Vancouver, BC; Faculty of Medicine, University of British Columbia, Vancouver, BC; Division of Pediatric Plastic Surgery, British Columbia Children's Hospital, Vancouver, BC; Division of Plastic Surgery, University of Birtish Columbia, Vancouver, BC; Faculty of Medicine, University of British Columbia, Vancouver, BC; Division of Pediatric Plastic Surgery, British Columbia Children's Hospital, Vancouver, BC

## Abstract

**Introduction:**

Children undergoing acute burn care may experience pain and anxiety affecting their ability to tolerate wound care procedures. Non-pharmacological support, including Child Life Specialists (CLS), may compliment pharmacological treatments to reduce pain and anxiety. CLS support is particularly relevant in pediatric burn care; however, there is a paucity of literature on the impact of CLS interventions for pediatric burn care. This review aims to synthesize the current understanding of CLS in pediatric burn care.

**Methods:**

A systematic review of peer reviewed databases (Embase, CINAHL, MEDLINE, Scopus) and gray literature (Google Scholar, Proquest, Papers First) was conducted up until 28 April 2023. Search terms included ‘burn care’ and ‘child life specialist*.’ Included studies focused on acute burn care and patient centered outcomes as primary outcomes, and non-clinical studies, such as reviews, were excluded. The protocol was published on PROSPERO, and PRISMA guidelines were followed. Data on study type, burn characteristics, pain, and anxiety were extracted and analyzed descriptively.

**Results:**

Seventy-one unique studies were found. Five publications (three peer reviewed articles and two conference abstracts) were included. Across the five included publications (n=226) patient median/mean age ranged from 2.2 years to 7.8 years old. Two studies had a control group (n=121). There was incomplete reporting of burn characteristics. Mean/median TBSA ranged from 0.5% to 9.17%, with the upper extremity the most frequently reported location (up to 66.7%). CLS interventions included inpatient and outpatient settings. Three studies reported pharmacological analgesia and CLS during burn care. CLS interventions included distraction with electronics and/or toys (4/5), music (1/5), and directed play (1/5). Patient and parent perceptions of pain and anxiety were collected via validated measures. For the two studies with control groups, CLS interventions were associated with a reduction in pain. There was no statistically significant reduction in anxiety.

**Conclusions:**

This systematic review identified few published studies on the effect of CLS interventions in pediatric burn care, with heterogeneity in patient population and outcome measures. CLS interventions were associated with reduced pain in children undergoing acute burn care. Although we did not find a statistically significant reduction in anxiety, authors reported insufficient statistical power.

**Applicability of Research to Practice:**

Preliminary research supports the role of CLS interventions in reducing pain in pediatric burn care. Higher powered studies examining the role of CLS in pediatric burn care are needed to further elucidate the effect of CLS on pain and anxiety. We hope these findings stimulate further research to provide the evidence needed to fund CLS as a permanent member of multidisciplinary pediatric burn care teams.

